# Quality indicators for collaborative care networks in persistent somatic symptoms and functional disorders: a modified delphi study

**DOI:** 10.1186/s12913-024-10589-w

**Published:** 2024-02-21

**Authors:** Nick Mamo, Lineke M. Tak, Manouk A. W. van de Klundert, Tim C. Olde Hartman, Judith G. M. Rosmalen, Denise J. C. Hanssen

**Affiliations:** 1 Department of Psychiatry, University of Groningen, University Medical Center Groningen, Groningen, Netherlands; 2 Dimence Institute for Specialized Mental Health Care, Alkura Specialist Center Persistent Somatic Symptoms, Deventer, Netherlands; 3 Department of Public Health, Faculty of Health and Medical Sciences, University of Copenhagen, Copenhagen, Denmark; 4 Department of Primary and Community Care, Radboud University Medical Center, Research Institute for Medical Innovation, Nijmegen, Netherlands; 5 Department of Internal Medicine, University of Groningen, University Medical Center Groningen, Groningen, Netherlands

**Keywords:** Collaborative care, Functional disorders, Persistent somatic symptoms, Quality indicators

## Abstract

**Background:**

Care for persistent somatic symptoms and functional disorders (PSS/FD) is often fragmented. Collaborative care networks (CCNs) may improve care quality for PSS/FD. Effectiveness likely depends on their functioning, but we lack a straightforward quality evaluation system. We therefore aimed to develop quality indicators to evaluate CCNs for PSS/FD.

**Method:**

Using an online three-round modified Delphi process, an expert panel provided, selected and ranked quality indicators for CCNs in PSS/FD. Recruited experts were diverse healthcare professionals with relevant experience in PSS/FD care in the Netherlands.

**Results:**

The expert panel consisted of 86 professionals representing 15 disciplines, most commonly physiotherapists, psychologists and medical specialists. 58% had more than 10 years experience in PSS/FD care. Round one resulted in 994 quotations, which resulted in 46 unique quality indicators. These were prioritised in round two and ranked in round three by the panel, resulting in a final top ten. The top three indicators were: “shared vision of care for PSS/FD”, “pathways tailored to the individual patient”, and “sufficiently-experienced caregivers for PSS/FD”.

**Conclusions:**

The identified quality indicators to evaluate CCNs in the field of PSS/FD can be implemented in clinical practice and may be useful in improving services and when assessing effectiveness.

**Supplementary Information:**

The online version contains supplementary material available at 10.1186/s12913-024-10589-w.

## Background

Functional disorders (FD), a group of disorders recognised by characteristic patterns of somatic symptoms, are a distinct part of the broader group of persistent somatic symptoms (PSS) [1]. It is generally assumed that the emergence and perpetuation of these conditions involve an interplay of multiple biopsychosocial factors [2]. This complexity results in people with both PSS and FD having long diagnostic delays, and may never receive the recommended treatment [3]. PSS and FD are also associated with a significant economic burden– due to both direct costs, such as medical appointments and investigations, and indirect costs, such as work absence [4, 5].

Care needs to be comprehensive and considerate of the whole person to provide good outcomes. Unfortunately, care systems are generally fragmented and specialised with notable limitations in the communication and collaboration of the involved disciplines [6]. This results in unnecessary medical investigations and high costs. Care fragmentation also impacts the care experience. Patients may experience long, and at times ‘aborted’ care trajectories, where they do not follow through on the complete care process. They also experience communication gaps across and within services. This results in poor outcomes as well as confusion and distrust [7]. Good PSS/FD care requires multidisciplinary involvement [8], preferably as collaborative care, as has been stated in research and commissioning guidelines [9]. The need for multidisciplinary involvement and collaborative care is due both to the earlier mentioned fragmentation of care, as well as the ingrained separation of mind and body in healthcare [6]. This also makes such multidisciplinarity and collaborative care inherently challenging to achieve.

One possible route towards improving this fragmentation is through collaborative care networks (CCN). The basic definition of a CCN is one where two or more health or social care professionals work together to provide care. This differs from multidisciplinary care in that multidsciplinary care does not necessary include collaboration between professionals. A CCN can range from professionals providing care for single patients, to a large regional network of multiple professionals directly or indirectly providing care for a specific group of patients. By focusing on improving the communication and collaboration of those involved in the management of PSS/FD, there is the potential to improve patients’ health outcomes. This improvement in outcomes could be achieved, among a number of ways, through better understanding of patients’ as well as professionals’ needs, and better access to care [10]. However, more systematic evidence is needed to show the effectiveness of such CCNs, while avoiding the assumption that collaboration is a positive outcome in itself [11].

CCNs in the care of FD are diverse, including in their size, involved disciplines and treatment options [12], making them complex interventions. Providing suitable quality indicators would allow for assessment and improvement of the quality of a CCN for PSS/FD, which may lead then to better outcomes from that CCN. Quality indicators are tools or flags that allow for monitoring and evaluation of clinical support services and organisational function towards improving patient outcomes [13]. Quality indicators can refer to structural or process elements of the networks, or relate to network outcomes [14]. Using this categorisation would provide more understanding of the types of indicators identified, and may assist in better application and evaluation in practice through matching indicators to evaluation methods. By using quality indicators, quality of care can be documented, compared across different locations or time-points, and can be used for priority setting within services. Since quality indicators for CCNs in PSS/FD care have to the best of our knowledge not been studied systematically [15], a consensus-based approach involving a panel of experts [13] would be a first step towards assessing and improving CCNs for PSS/FD.

In this study, we aim to develop a prioritised and ranked list of quality indicators that are realistic and representative of the experiences of professionals working in PSS and FD care.

## Methods

The current study is part of the innovative training network ETUDE (Encompassing Training in fUnctional Disorders across Europe; etude-itn.eu), ultimately aiming to improve the understanding of mechanisms, diagnosis, treatment and stigmatisation of FD [1]. The study was registered on the Open Science Framework (OSF - osf.io/f9d5x).

### Study design

In order to identify relevant quality indicators for CCNs in PSS/FD, a three-round modified Delphi study was designed. The Delphi method is an iterative process to find consensus on a subject among a group of experts through a combination of idea generation, prioritisation and ranking over multiple rounds [16]. Aside from the iterations of questionnaires with controlled regular feedback, the quality of the Delphi method is also maintained through the pseudonymity of panel members [17].

It is important to mention one significant change from the original study plan described in the study registration. The original aim was to have three expert panels - one in the UK, one in Germany and one in the Netherlands - with a minimum of 30 in each. As the study progressed, we got a much larger number than expected in the Netherlands, however well below the minimum required numbers in the UK and Germany. For this reason, it was decided to stop the UK and Germany arms of the study and concentrate instead on the Netherlands.

### Expert panel

Eligible respondents for the expert panel were defined as registered health or social care professionals with experience in CCNs providing care for PSS/FD. While there is no agreed number of respondents in a Delphi study, the majority of studies have a panel of between ten and 100 experts, with 30 to 50 being most often recommended [17, 18]. Alongside this, a level of attrition of respondents between rounds is accepted. In line with the literature, we aimed for a maximum of 30% loss between rounds to maintain rigour and demonstrate consensus across the panel [19].

Invitations to participate in the Delphi study were sent in an open call through relevant mailing lists and contacts identified by the research team. This included professional organisations such as the Dutch national network on PSS (NALK, www.nalk.info) as well as informal networks and personal contacts. During round one participants were provided with definitions of CCN and PSS/FD, clarifying that we were seeking respondents in this field. During this round we also asked about length and field of experience.

As an incentive, we offered each participant from the expert panel to donate money to a charity of their choice; one euro per respondent in round one, two euros in round two, and three euros in round three.

Information was provided on the use and storage of personal data in the information letter and invitation email, alongside consent questions, in accordance with Dutch and EU law.

### Procedure and analysis

#### Round 1: idea generation

The aim of round one was pseudonymous idea generation on indicators of quality of CCNs for PSS/FD care by a panel of experts. In this round, relevant experts received an email invitation with an information sheet explaining the aim of the study and a link to an online questionnaire designed on Google Forms (see Appendix 1 for details– questionnaire designed for this study). Questions included general characteristics such as profession, setting, years of work experience and the main patient group served (such as general population, functional neurological disorder or others). The focus in this round was to draw out ideas and identify perspectives on quality of care in a CCN for PSS/FD. We asked this in three open questions: ‘how can you tell if a network is giving good results?’; ‘what characteristics in a care network can be used to track service quality?’; and ‘how can a network demonstrate that the processes in place are working?’. With the first question we provided no examples. On the next page, three examples were provided for the second question and two examples were provided for the third question. The combination of no examples with the first question, and examples in the following questions was to provide a balance between ensuring these questions were understood while avoiding influencing the answers by providing more information than necessary.

We provided the following definition of a CCN to the expert panel as part of the online questionnaire:

*“Collaborative care involves providers from different specialties, disciplines or sectors working together to offer complementary services and mutual support, to ensure that individuals receive the most appropriate service from the most appropriate provider in the most suitable location, as quickly as necessary, and with a minimum of obstacles. Collaboration can involve better communication, closer personal contacts, sharing of clinical care, joint educational programs and/or joint program and system planning”* [20].

The results from the idea generation round were independently coded by two coders (LT - psychiatrist and head of a tertiary service for patients with PSS/FD - and MK - a masters student and expert by experience) for increased reliability. Coding was done using ATLAS.ti, a qualitative analysis software programme, where the data was directly uploaded following extraction, with no modifications needed. A third coder (NM -general practitioner and PhD candidate focusing on collaborative care in PSS/FD) reviewed for consensus, also providing an initial code list of possible quality indicators for CCNs in PSS/FD care based on the initial results from the incomplete UK arm of the study. Coding was undertaken inductively, as well as deductively using the initial code list.

The initial list of possible quality indicators was reviewed by four of the authors (DH, JR, LT, NM), with an iterative process of reviewing and refining them. Quality indicators that were only rarely mentioned (by maximum two experts) were removed from the this list. Fifty-three indicators were removed as they were mentioned by only one or two experts, as an initial step to select the most relevant indicators while keeping the complete list feasible for the selection process in round 2.

#### Round 2: narrowing down

The complete list of 46 quality indicators was then presented to the expert panel, for which they were asked to select the ten quality indicators they considered to be most important in the evaluation of a CCN for PSS/FD. The ten indicators that were selected most often were taken as the top ten indicators over all. A maximum of two reminders were sent to each expert to complete the questionnaire. Both round two and three were run through Qualtrics, sending questionnaire links to all respondents from round one.

#### Round 3: ranking

The final list of ten quality indicators was again presented to the expert panel. This time, the expert panel was asked to rank these from most important (position 1) to least important (position 10). Ranking options were restricted so that it was not possible to assign the same rank to more than one quality indicator. A maximum of two reminders were sent to each expert to complete the questionnaire. The overall final ranking was based on the mean rank scores provided automatically by the Qualtrics platform. Data collection for all three rounds was conducted between January and December 2022.

The top ten quality indicators were then assigned as either structure, process or outcome indicators [14] by three of the authors (DH, JR and NM).

## Results

### Participants

Of the 111 respondents, 86 were considered eligible and thus formed the expert panel. The 25 exclusions did not have registered health or social care professions, but included massage therapists and lifestyle coaches. Table 1 gives an overview of the characteristics of the expert panel. The vast majority of respondents were women (88%), and 58% had more than 10 years of experience working with patients with PSS/FD. A number of disciplines were included, with physiotherapists, psychologists and medical specialists (including psychiatrists) representing the largest numbers. However, there were also general practitioners, exercise therapists, and others such as nurses, an occupational therapist and one person from a social care background.

Of the complete eligible expert panel of 86 included in round 1, 85% (*n* = 73) responded in round 2 and 74% (*n* = 64) responded in round 3.

**Table 1:** Expert panel demographics

*Total: 86*	
**Female -** ***N*** **(%)**	76 (88)
**Age - Mean (SD)**	47.02 (9.7)
**Profession -** ***N*** **(%)**	
Psychologist	15 (17)
Physiotherapist	28 (33)
General Practitioner	6 (7)
Medical specialist	15 (17)
Exercise therapist	11 (13)
Other	11 (13)
**Years of experience in the field -** ***N*** **(%)**	
Less than a year	1 (1)
1–2 years	5 (6)
3–5 years	14 (16)
5–10 years	16 (19)
more than 10 years	50 (58)
**Workplace -** ***N*** **(%)**	
1º care	46 (53)
2º care	24 (28)
3º care	9 (11)
Other	7 (8)
**Served patient population -** ***N*** **(%)**	
PSS/FD (adult)	40 (47)
Chronic pain (adult)	17 (20)
Mental health (adult)	5 (6)
General (adult)	5 (6)
PSS/FD (children and adolescents)	11 (13)
General (children and adolescents)	5 (6)
Other	3 (3)

Abbreviations: PSS - Persistent somatic symptoms; FD - functional disorders

### Quality indicators for CCNs in PSS/FD care

In total, 994 ideas for potential quality indicators were provided by the 86 experts participating in round 1, with the minimum and maximum number of ideas per participant being 2 and 23. From these, 99 unique indicators were identified after removing overlap and similarity. Fifty-three indicators were removed as they were mentioned by only one or two experts. This left a list of 46 quality indicators that were presented to the expert panel in round 2 (see Appendix 2).

Table 2 shows the combined results of rounds 2 and 3, by showing the top ten indicators. As can be seen here, there is close consensus between rounds two, where the top ten were selected, and round three, where the top ten indicators were ranked. This showed that the rate at which quality indicators were selected in round two was reflected in the overall ranking from round three. We do note some changes in order, however. For example, the third most selected indicator in round two - “Open communication between healthcare providers” - is ranked fifth in round three. Conversely, the seventh most selected indicator in round two - “Sufficiently-experienced caregivers for PSS/FD” - was ranked third in round three.

**Table 2:** Selected (round 2) and ranked (round 3) quality indicators for CCNs in the field of PSS/FD

Rank	Quality Indicators for CCNs for PSS/FD	*Round 2 (n = 73)*	*Round 3 (n = 64)*
		% (*n*) of experts selecting indicator	mean rank (SD)
1	Shared vision of care for PSS/FD	6.6% (44)	3.7 (2.8)
2	Pathways tailored to the individual patient	5.4% (36)	4.3 (2.6)
3	Sufficiently-experienced caregivers for PSS/FD	4.2% (28)	4.8 (2.9)
4	Shared decision-making with patients	4.0% (27)	5.1 (2.6)
5	Open communication between healthcare providers	5.0% (34)	5.3 (2.3)
6	Awareness of the expertise of other disciplines	4.9% (33)	5.8 (2.8)
7	Multidisciplinary consultation	4.9% (33)	6.1 (2.6)
8	Acceptable waiting times for intake, diagnosis and treatment	4.3% (29)	6.5 (2.9)
9	Multidisciplinary involvement in diagnostics	3.7% (25)	6.6 (2.8)
10	Active collaboration with somatic specialists	3.7% (25)	6.7 (2.7)

Abbreviations: CCN - collaborative care network, FD - functional disorders, PSS - persistent somatic symptoms, SD - standard deviation.

The different quality indicators represent varying levels of complexity and nuance. Examples illustrating each are shown in Table 3. In some cases, the quality indicator used summarises quite homogeneous quotations, while others represent more heterogeneous codes, with a wider range of perspectives. Examples of narrow-focus quality indicators include “Sufficiently-experienced caregivers for PSS/FD” and “Pathways tailored to the individual patient”. The most notable example of an indicator with heterogeneous codes is “Open communication between healthcare providers”. The quotations under this indicator can be split into: clear communication, good communication, open communication, regular sharing, communication systems, findability, and short lines/efficient communication.

By grouping the indicators into structural, process and outcome indicators [14], the indicators here fall into two of the categories - structure and process. Structural indicators included three indicators, while process indicators included seven. No outcome indicators were selected amongst the top ten at the end of round two. Of the five outcome indicators originally listed at the end of round one, the highest-selected at the end of round two - *“Evaluation of patient satisfaction”* - was only selected by 15 experts, with 18 other indicators selected more often.

**Table 3:** Illustrative quotations for final quality indicators for CCNs for PSS/FD

Quality Indicator	Structure/ Process/ Outcome	Ideas
**Shared vision of care for PSS/FD**	**Structure**	*“Client experiences network of care rather than separate caregivers who seem to contradict each other”*
		*“Shared vision”*
		*“It is important that the disciplines within the network deal with the client’s complaints in the same way, i.e. all being on the same page and coming across as one to the client”*
**Pathways tailored to the individual patient**	**Process**	*“Presence of care plan or care map”*
		*“Is customization possible or only standard care pathways etc.”*
		*“There is room to make adjustments to treatment goals during treatment.”*
**Sufficiently-experienced caregivers for PSS/FD**	**Structure**	*“Knowledge and experience in the field of PSS”*
		*“Caregivers are competent”*
		*“Expertise”*
**Shared decision-making with patients**	**Process**	*“Is the ‘Deciding Together’ model being used?”*
		*“Information exchange regarding treatment methods, but also reactions of clients”*
**Open communication between healthcare providers**	**Process**	*“Sincerity”*
		*“Open culture so that questions dare to be asked and this is encouraged that repeated consultation/questioning is okay and allowed”*
		*“Referrals and feedback to each other”*
		*“Sharing knowledge and experiences”*
		*“Way of communicating is established”*
		*“Short lines”*
**Awareness of the expertise of other disciplines**	**Structure**	*“If a healthcare network knows very well what they can and cannot treat”*
		*“Knowing each other allowing you to coordinate care in advance in a care pathway”*
		*“Easy transfer of patients if another treating colleague can do it better”*
		*“Being well-versed in the expertise of colleagues in the network”*
**Multidisciplinary consultation**	**Process**	*“Joint treatment evaluation”*
		*“Multi- / interdisciplinary patient consultation”*
		*“Being able to work in a multidisciplinary way also in the first line with colleagues who also have knowledge of PSS. Because there is more knowledge, treatment can get off the ground faster and healthcare providers work together more efficiently.”*
**Acceptable waiting times for intake, diagnosis and treatment**	**Process**	*“Decrease wait time to intake, wait time to diagnosis and advice and wait time to initial treatment”*
		*“Waiting lists for the various links in the care chain”*
		*“Pathway of diagnostics takes shorter time”*
		*“Rapid start of treatment after diagnosis of PSS”*
**Multidisciplinary involvement in diagnostics**	**Process**	*“Interdisciplinary diagnostics”*
		*“A multidisciplinary intake with physiotherapists, occupational therapist, medical social worker and a psychologist - and on indication a speech therapist, a dietician, etc.”*
		*“Deploy a rapid broad intake and help in multiple areas simultaneously”*
**Active collaboration with somatic specialists**	**Process**	*“Connecting to treatment in specific PSS clinics/teams”*
		*“Neurologist who is easily approachable”*

Abbreviations: CCN - collaborative care network, FD - functional disorders, PSS - persistent somatic symptoms

## Discussion

### Principal findings

Through a modified Delphi approach with a broad, 86-member expert panel, we successfully identified and ranked ten quality indicators for CCNs in PSS/FD care from a developed list of 46. Of these indicators, the highest ranked are “Shared vision of care for PSS/FD”, “Pathways tailored to the individual patient” and “Sufficiently-experienced caregivers for PSS/FD”. The final list of quality indicators offers a realistic, nuanced view of how a CCN in PSS/FD care should look according to healthcare professionals, prioritising coherent care, with a patient-centred approach that is connected to other relevant health and social care services.

### Strengths and limitations

This study has a number of strengths. The choice of a modified Delphi has the strength of providing ecological validity, with results that are relevant to currently-active health and social care professionals who may now or in the future be active in a CCN.

Methodologically speaking, the Delphi process provides an aggregate of individual opinions to reduce biases from dominant individuals, group pressure and irrelevant communication [17, 19]. The rigour of the current study is shown in part by the coding procedure with two independent coders of different and relevant backgrounds, and an iterative reviewing process. Methodological strength is also shown by a large and diverse expert panel, with a wealth of experience in the field of PSS/FD CCNs. We also maintained a large proportion of the expert panel between rounds with a drop-out rate of less than 30%. There is robust consensus demonstrated in rounds two and three with strong concurrence in results between the two rounds. However, the standard deviations in round three are quite wide. This suggests that the final ranking may be of lower importance than the selection of which are the top ten indicators.

Some other study limitations have to be discussed. Firstly, as mentioned, the original plan of undertaking this study in three countries had to change to focus on only one country. Unfortunately, this means that no comparison could be drawn between different countries. This also means that the results of this study are specific to the Netherlands, and application in other settings must be undertaken with caution, though the results would likely be similar.

Another principal weakness of this study is that we did not include any patients or experts by experience, for whom CCNs are designed. This was not within the primary scope of the study, however, patient involvement is an important next step. This would be important to challenge the results and further increase external validity through the perspective of those accessing the services of a CCN for PSS/FD. It is also noted that we only had one person from a social care background. Better representation from this field may have influenced our results, as well as increasing validity by better reflecting the professionals involved in patient care.

In addition, while quality indicators for CCNs are preferably specific and directly measurable [13, 15], the indicators we defined may seem too non-specific. This does pose some challenges since applying them in practice will be more complex than would have been the case if more specific and directly measurable indicators had been prioritized. However, their broader applicability can be a strength, allowing for localised, needs-based application. This is especially important considering the principles of a CCN, which can look very different depending on its setting and aims. Indeed, CCNs for FD differ significantly in team size and make-up, treatment modalities offered, and the areas of care in which the teams collaborate [12]. In view of this, broader defined quality indicators provide the opportunity for tailoring these indicators to the characteristics of specific PSS/FD CCNs. Related to this is the lack of outcome indicators, which may be a result of the phrasing of the questions asked, or reflects the priorities of the expert panel.

### Comparison with the literature

While this is the first study we are aware of to look at quality indicators for CCNs for PSS/FD, other studies have looked at quality indicators in CCNs in other fields - whether through systematic review, Delphi study, or other methods [21–25].

Similar to the present study, the results of these studies include a mixture of structure and process indicators. However, some also include a number of outcome indicators that are often quite specific, such as the percentage of persons with a specific diagnosis in a study on indicators in integrated care for schizophrenia (22). Although we see some notable differences in quality indicators compared to this study, in others we see significant agreement, with many studies showing similar findings to the present study. These include shared mission and vision among professionals, knowledge of interprofessional team roles and contributions, and interprofessional and informal communication in a study on end-of-life care [23]. Shared decision-making and acceptable waiting times, and the presence of other disciplines in multidisciplinary team meetings were identified in a study on head and neck cancer [22]. In another study on dementia care, we see coordination with external groups and collaboration with other providers, team-based care and dedicated care coordination, and shared decision-making [24]. A study on collaboration in post-surgery care included the perspective of patients and family, alongside team members [25]. Here, we see indicators of communication and developing conceptual alignment, insight into the expertise of other disciplines, as well as the importance of time-efficiency. The similarities between this study on post-surgery care and our study give the impression that adding patients to the expert panel would not have yielded additional results. In summary, the results of both our and previous studies identified indicators that are relevant across a wide range of CCNs, such as shared vision of care, knowledge of team members’ expertise, and open communication within the team.

### Implications for practice and future research

This study provides a list of the quality indicators considered most important by healthcare professionals working in CCNs for PSS/FD. This list can, therefore, be used by clinicians and policy-makers as a framework of priorities around which to build or strengthen such CCNs.

In the absence of reliable and valid tools to assess CCN function, the list can also be used as an initial assessment of how a network performs according to its members, in these different areas. In addition, it can be a starting point to use expert input to develop realistic strategies to improve CCN functioning. These strategies can be identified by methods such as Nominal Group Technique [26] or Research World Café [27].

The results are also important with regards to the challenge of providing evidence of effectiveness of complex interventions. Randomised controlled trials (RCTs) remain a very important way to test the effectiveness of an intervention, i.e. improve patient outcomes, professional outcomes, and cost-effectiveness. However, RCTs are limited in complex interventions - such as CCNs - as they would not be able to tell us which specific elements or changes are having an impact on outcomes. For this reason, alternative methods must be devised for CCNs. Such methods would allow us to gain insight into which characteristics of CCNs, and changes within these, are impacting on outcomes. The current quality indicators can be of great use in achieving this. As we have stated, the quality indicators are all structure or process indicators, with no outcome indicators. The questions asked may have suggested less focus on outcomes, however a number of outcome-focused indicators were listed originally, and not prioritised. Therefore, the reasons behind the lack of outcome indicators remain unclear, other than to say that it reflects the priorities of the expert panel. Nonetheless, the current quality indicators would need to be linked with relevant outcome measures to show the effectiveness of CCNs. A suitable group of outcome measures to combine with would be the quintuple aim - combining individual experience, population health, healthcare costs, experience of care providers and health equity [28]. It is also important to state that an evaluation plan to show CCN effectiveness would have to be specific to particular CCNs, and therefore cannot be generalisable to all CCNs. This reinforces the importance of adaptation of the application of the quality indicators to local contexts and needs. With this in mind, we can, as a starting point, use the indicators to measure and possibly improve the quality of CCNs for PSS/FD, for instance by conducting Participatory Action Research. From here, it may be possible to show the effectiveness of specific CCNs for PSS/FD by combining our structure and process indicators with outcome measures such as the quintuple aim. This avoids the problem of assessing the quality of CCNs by relying on collaboration as an outcome measure in itself, which assumes that improved collaboration automatically implies improved outcomes [11]. The combination of our quality indicators with the quintuple aim provides a potential alternative to RCTs by indicating which characteristics and changes are influencing the outcomes, whether by using Participatory Action Research or other methods.

Although many of these indicators may seem simple, they can have a profound positive impact if taken seriously, and a profound negative impact if ignored. The top indicator, “shared vision of care for PSS/FD” is a prime example of this. Many patients report receiving contradictory information from different healthcare professionals treating the same illness, resulting in a poor experience and distrust, and demonstrating a lack of shared vision [7]. Within the field of PSS/FD this is particularly important given the ongoing discussion on the different names, as well as the competing and varied explanatory models used [29, 30].

Further work is required to move from this list towards a realistic evaluation plan, in other words, ‘operationalising’ these indicators. Not all these indicators will necessarily apply to all CCNs for PSS/FD - this will depend on the aims and focuses of different networks. As an example, waiting times may not be directly relevant to a network that does not directly provide patient care. We also argue that these issues are not limited to PSS/FD care. Therefore, these indicators can be applied with appropriate modification, in other fields, such as multimorbidity where complex conditions require complex interventions.

### Conclusions

The results of this study - ten quality indicators for CCNs in PSS/FD - are an important step towards dealing with the issues caused by the fragmentation of care in this field, and the poor quality of care experienced. The quality indicators can be applied in different contexts, tailored for specific CCNs in PSS/FD care. The connection between improved CCNs for PSS/FD through these indicators, and improved patient outcomes and satisfaction has yet to be studied. This has the potential to provide evidence of effectiveness of specific CCNs - services built around collaboration, tailored to the needs of a local population.

### Electronic supplementary material

Below is the link to the electronic supplementary material.

Supplementary Material 1

Supplementary Material 2

## Data Availability

Registration and further data can be found on the OSF platform here: osf.io/f9d5x.

## Abbreviations

CCN: Collaborative care network
ETUDE: Encompassing Training in fUnctional Disorders across Europe
FD: Functional disorders
NALK: Netwerk Aanhoudende Lichamelijke Klachten (Dutch national network on persistent somatic symptoms)
OSF: Open science framework
PSS: Persistent somatic symptoms
RCT: Randomised controlled trial
