# Supplementary material for: Quality indicators for collaborative care networks in persistent somatic symptoms and functional disorders: a modified delphi study
Source: BMC Health Serv Res. 2024 Feb 21;24:225. doi: 10.1186/s12913-024-10589-w (PMC10882926; doi:10.1186/s12913-024-10589-w)
Supplement: Supplementary file 1 — Supplementary Material 1 [file 12913_2024_10589_MOESM1_ESM.docx]

**Appendix 1 - Round 1 Questionnaire**

**Collaborative Care Networks - Quality Indicators**

This is Round One in a Delphi study to determine important quality indicators for a collaborative care network in the management of functional disorders and persistent somatic symptoms (anything from bromyalgia to psychogenic non-epileptic seizures (PNES) to chronic pain). You have been sent this form because we think that you are someone with experience working together with other professionals in providing care for people with such conditions, and so we believe that your input is valuable. We would appreciate any time and ideas you could share with us.

What do we mean by a collaborative care network in functional disorders and persistent somatic symptoms? Broadly speaking, a collaborative care network is where two or more health or social care professionals work together to provide care to persons with functional disorders or persistent somatic symptoms.

It may also be useful to consider this: "Collaboration can involve better communication, closer personal contacts, sharing of clinical care, joint educational programs and/or joint program and system planning"(Craven, 2006).

In this round what we are looking for is any and all ideas. No idea too big or too small. If you're not sure it's important, please do write it anyway.

* Indicates required question

1. Email *
2. Do you consent to being included in this study? * *Mark only one oval.*

- Yes

1. Do you consent to be contacted if there are further questions related to this study, including a follow-up to this study? *Mark only one oval.*

- Yes to any further involvement
- Yes but only to questions on this study
- No

Section 1 of 3 - Demographics

1. Age*
2. Gender *
   *Mark only one oval.*

- Woman
- Man
- Non-Binary
- Prefer not to say
- Other:

1. Profession * *Mark only one oval.*

- Nurse
- Physiotherapist
- Doctor (GP)
- Doctor (Specialist)
- Psychologist
- Social worker
- Social Prescriber/Links worker
- Occupational Therapist
- Mental Health worker
- Pharmacist
- Other:

1. Setting * *Mark only one oval.*

- Primary Care/GP Practice
- Secondary Care/Hospital
- Secondary Care/Other
- Private practice
- Other:

1. If relevant: in which department do you work?
2. City/Region *
3. How long have you been providing care for persons with Functional * Disorders/Persistent somatic symptoms (FD/PSS)? *Mark only one oval.*

- Less than a year 1-2 years
- 3-5 years
- 5-10 years
- more than 10 years

1. Describe the main patient group your network services are offered to
2. As a thank you for taking part in the study, we would like to donate €1 to charity for every respondent. Please choose which charity you would like us to donate to on your behalf. *Mark only one oval.*

- Red Cross (icrc.org) - support and medical care in conflict zones
- Cool Earth (coolearth.org) - climate activism, working with indigenous communities to protect the rainforests
- Partners in Health (pih.org) - creating and supporting sustainable health systems in low-resource settings

Section 2 of 3 - Quality Indicators - General

Here, please write any and all ideas that you have. No idea too big or too small. If you're not sure it's important, please do write it anyway.

1. How can you tell if a network is giving good results?

What measures or markers might you use to demonstrate that a network is working well? Try to be specific.

Section 3 of 3 - Quality Indicators 2

Again, please write any and all ideas that you have. No idea too big or too small. If you're not sure it's important, please do write it anyway.

Here we will provide you with two questions and some associated examples to help with considering other options

1. What characteristics in a care network can be used to track service quality? Try to be specific.

Possible examples: frequency of network meetings; disciplines involved in the network; sucient consultation space.

1. How can a network demonstrate that the processes in place are working?

Possible examples: reductions in referral waiting times; reductions in time to diagnosis

Any other ideas...[in case you have run out of space]
