# Supplementary material for: Quality indicators for collaborative care networks in persistent somatic symptoms and functional disorders: a modified delphi study
Source: BMC Health Serv Res. 2024 Feb 21;24:225. doi: 10.1186/s12913-024-10589-w (PMC10882926; doi:10.1186/s12913-024-10589-w)
Supplement: Supplementary file 2 — Supplementary Material 2 [file 12913_2024_10589_MOESM2_ESM.docx]

**Appendix 2 - Final code list with ranking order from round 2**

| **Codes** | Choice count |
| --- | --- |
| Shared vision of care for persistent somatic symptoms | 44 |
| Pathways tailored to the individual patient | 36 |
| Open communication between healthcare providers | 34 |
| Awareness of the expertise of other disciplines | 33 |
| Multidisciplinary consultation | 33 |
| Acceptable waiting times for intake, diagnosis and treatment | 29 |
| Sufficient experienced caregivers for persistent somatic symptoms | 28 |
| Shared decision-making with patients | 27 |
| Active collaboration with somatic specialists | 25 |
| Multidisciplinary involvement in diagnostics | 25 |
| Clear overview of treatment options | 23 |
| Accessible consultation possibilities | 20 |
| Consistent cross-disciplinary use of explanatory models | 18 |
| Involvement of the social domain | 18 |
| Regular regional network meetings | 17 |
| Agreement on division of roles in network | 16 |
| Joint education for health care providers | 16 |
| Initiatives for innovations | 16 |
| Cross-disciplinary use of consistent terminology | 15 |
| Availability of a regional care map | 15 |
| Evaluation of patient satisfaction | 15 |
| Cross-disciplinary availability of patient education materials | 14 |
| Agreements on guidelines and standards of care to be used | 14 |
| User-friendly communication system | 14 |
| Clarity about reimbursement | 12 |
| Agreements on referral procedures | 12 |
| Agreements on communication around transition moments | 11 |
| Network-wide access to medical records | 11 |
| Presence of informal contacts | 10 |
| Evaluation of health-related quality of life in patients | 8 |
| Cross-disciplinary explicit treatment goals | 8 |
| Number of joint cross-disciplinary consultations | 7 |
| Network has process in place to identify gaps in care delivery | 6 |
| Clarity on cross-disciplinary care coordinator | 6 |
| Number of disciplines represented | 5 |
| Evaluation of referral satisfaction | 5 |
| Evaluation of regional network functioning | 4 |
| Network-wide availability of e-health options | 3 |
| Evaluation of utilization of care | 3 |
| Number of referrals to healthcare providers in the network | 3 |
| Evaluation of satisfaction of close relatives patient | 3 |
| Possibility for patients to access their medical records | 3 |
| Regular audits of quality of care | 2 |
| Presence of information on waiting times | 1 |
| Evaluation of caregiver job satisfaction | 1 |
| Rate of caregiver attrition and absenteeism | 1 |
